# Army Medical Board of Examiners

**Published:** 1861-05

**Authors:** 


ARMY MEDICAL BOARD OF EXAMINERS.—Surgeons C. A. Finley, C. McDougall, and W. J. Sloan will meet in New York City on the first of May, or as soon thereafter as practicable, for the purpose of examining assistant surgeons for promotion, and such candidates for admission into the army medical staff as may have obtained permission from the Secretary of War.

